# The Implementation of Pharmacy Competence Teaching in Estonia

**DOI:** 10.3390/pharmacy5020018

**Published:** 2017-03-31

**Authors:** Daisy Volmer, Kristiina Sepp, Peep Veski, Ain Raal

**Affiliations:** 1 Institute of Pharmacy, University of Tartu, Nooruse 1, 50411 Tartu, Estonia; ain.raal@ut.ee (A.R.); 2 Estonian Pharmacies Association; Vae 16, Laagri, 76401 Harju County, Estonia; kristiina.sepp@apotheka.ee

**Keywords:** competence, competency, pharmacy education, Estonia

## Abstract

**Background:** The PHAR-QA, “Quality Assurance in European Pharmacy Education and Training”, project has produced the European Pharmacy Competence Framework (EPCF). The aim of this study was to evaluate the existing pharmacy programme at the University of Tartu, using the EPCF. **Methods:** A qualitative assessment of the pharmacy programme by a convenience sample (*n* = 14) representing different pharmacy stakeholders in Estonia. EPCF competency levels were determined by using a five-point scale tool adopted from the Dutch competency standards framework. Mean scores of competency levels given by academia and other pharmacy stakeholders were compared. **Results:** Medical and social sciences, pharmaceutical technology, and pharmacy internship were more frequent subject areas contributing to EPCF competencies. In almost all domains, the competency level was seen higher by academia than by other pharmacy stakeholders. Despite on-board theoretical knowledge, the competency level at graduation could be insufficient for independent professional practice. Other pharmacy stakeholders would improve practical implementation of theoretical knowledge, especially to increase patient care competencies. **Conclusions:** The EPCF was utilized to evaluate professional competencies of entry-level pharmacists who have completed a traditional pharmacy curriculum. More efficient training methods and involvement of practicing specialists were suggested to reduce the gaps of the existing pharmacy programme. Applicability of competence teaching in Estonia requires more research and collaborative communication within the pharmacy sector.

## 1. Introduction

### 1.1. Pharmacy Education and Training in Estonia

**Figure 1:**
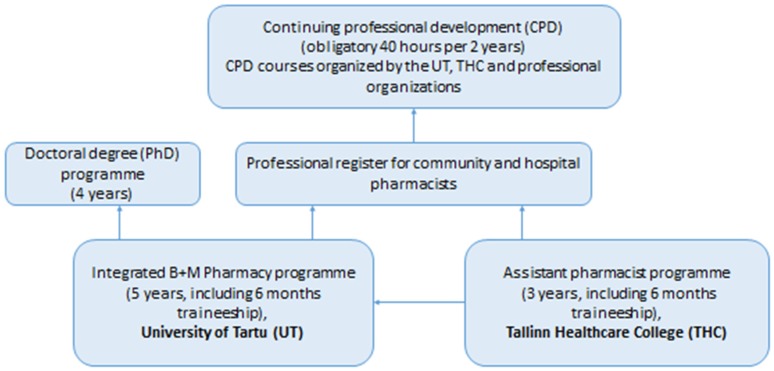
The pharmacy education and continuing professional development scheme in Estonia.

The Faculty of Medicine at the University of Tartu (UT), Estonia, provides higher education and several PhD programmes in medicine, dentistry, pharmacy, sport sciences, and physiotherapy [1]. Pharmacists (*proviisor* in Estonian) study at the UT for five years and graduate with a Masters of Pharmacy (MSc Pharm) (Figure 1). Pharmacists can be owners and managers of community pharmacies, work as responsible pharmacists in the community and hospital pharmacies, or other pharmacy fields (e.g., wholesale companies or in the pharmaceutical industry). Effective as of 2020, the ownership of community pharmacies will be limited to the pharmacy profession (more than 50 per cent of the shares have to belong to pharmacists). Currently, the majority of community pharmacies have joined different pharmacy chains and mainly traditional services (e.g., dispensing and counselling of the use of prescription and non-prescription medicines) have been provided. In addition, different extended services (e.g., diagnostic clinical tests) have developed [2]. Assistant pharmacists (*farmatseut* in Estonian) study at Tallinn Healthcare College for three years and are mainly employed in community pharmacies after graduation [3]. Specialists with a diploma of assistant pharmacist who want to continue their education and become pharmacists have to pass the full five-year programme at UT. All practicing pharmacists and assistant pharmacists have to be registered at the corresponding professional register and have to participate in the continuing professional development (CPD) courses for at least 40 h over two years [4] (Figure 1).

The pharmacy programme at UT is designed as medical subject-based and pharmaceutical product-oriented, following the sectorial profession model and the EU directives [5,6]. The curriculum is organized as a course based on five years of integrated bachelor and master training (300 ECTS). The existing pharmacy programme is the basis for recognition of professional qualification. Currently the pharmacy curriculum is available only in Estonian and does not offer specialization. However, international students could participate in research work or take single courses based on individual learning and examination [7]. 

The pharmacy programme is constantly reviewed and modified. The proportion of in-class lectures has decreased, while independent work has increased; problem-based and research-based training have been introduced in some subjects. The proportion of chemistry-based subjects has decreased and the amount of pharmaceutical technology and medical subjects has increased. A large proportion of medical subjects in the pharmacy programme was intended to prepare future pharmacists for more efficient collaboration in health care teams. During the last ten years, interdisciplinary subjects such as pharmacoeconomics and pharmacoepidemiology, biotechnology, genetics, and bioethics have been integrated into the programme. The patient care concept has been introduced by subjects such as primary care medicine, laboratory medicine, clinical microbiology, clinical pharmacology, and clinical pharmacy (the last subject was added in 2017) (see Appendix A). A comparison of the pharmacy programme of the UT with respective pharmacy curricula in the EU is presented in Table 1 [8].

In the Pharmacy programme, four subject groups—drug analysis (chemistry subjects and pharmacognosy), pharmaceutical technology, medical, and social sciences—have been identified (Table 2). Continuity of the subject areas is organized by the system of pre-subjects: subjects placed in the earlier years of training are pre-requisite subjects for the following, and more specialized, subjects (see Appendix A).

### 1.2. How to Achieve Competency Based Pharmacy Education in Estonia?

Current pharmacy programme at the UT is compiled using a traditional subject and course-based system. Identification and description of the “flow chart” of subject areas in the pharmacy programme could be seen as a start to describe the curriculum by competencies. The next possible step could be the connection of subject areas with particular entry-level pharmacist professional competencies in the future. 

Another possibility to collect valuable information for development of pharmacy training could be to use professional guidelines, e.g., “Community pharmacy service quality guidelines” [9] and “Estonian hospital pharmacy good practices” [10] that were developed and implemented in 2012 and 2015, respectively. Both guidelines shape the expected quality of pharmacy practice in community and hospital setting in Estonia. Community pharmacy guidelines list the indicators that every community pharmacy could use for self-assessment of its pharmacy operation and service provisions. Guideline based self-assessment reports have showed community pharmacists to have higher confidence about providing traditional rather than extended services. Patient-centred counselling of medicines and the provision of extended services are impeded by the shortage of professional personnel, the lack of private consultation possibilities at pharmacies, lack of motivation towards treatment, and use of medicines among patients, as well as by the insufficient professional competency of community pharmacists [2].

Changes within pharmacy profession competencies include moving from product- to patient-oriented knowledge and skills. More attention has to be paid to patient-centred pharmaceutical care services. There are several existing frameworks for evaluating changes in the performance and competence of practicing pharmacists [11,12,13,14,15,16]. For entry-level pharmacists, competency guidelines based on curriculum outcomes have been developed in Canada, Australia, and the UK [17,18,19]. The European Pharmacy Competence Framework (EPCF) has been produced within the project PHAR-QA, “Quality Assurance in European Pharmacy Education and Training”, as a new assessment tool for competency-based pharmacy training in Europe [20].

The aim of this study was to evaluate the existing pharmacy programme at the UT by using the EPCF:

- to construct a curriculum mapping matrix;
- to assess the pharmacy curriculum outcome based competency level, and
- to identify the pharmacy curriculum gaps and evaluate the expediency of the EPCF as a curriculum mapping tool.

## 2. Materials and Methods

### 2.1. PHAR-QA Framework

The EPCF was produced by EU project PHAR-QA, “Quality Assurance in European Pharmacy Education and Training”. Developed tool includes personal and patient care competences presented in 11 domains and 50 particularly defined competences (Appendix B). The tool has been validated in a two-round Delphi survey by more than 2000 representatives of different pharmacy stakeholders in Europe [21,22]. For the implementation of the EPCF, it was important to learn its usability as a guideline for evaluation and development of pharmacy curricula in the EU. UT, Institute of Pharmacy, acted as a partner in the PHAR-QA project, and volunteered to pilot the tool for the evaluation of the pharmacy programme. 

### 2.2. Study Design and Sample

The initial study design was developed and proposed by Prof. Andries Koster, University of Utrecht, Utrecht, Netherlands [23]. Among pharmacy schools piloting the EPCF, the following study structure was agreed upon:

- intended (existing) curriculum mapping: matrix construction of 50 EPCF competences and curriculum elements;
- evaluation of expected competency level at the graduation of first-degree curriculum (MSc degree); and
- identification of curriculum gaps.

A qualitative assessment of the pharmacy programme was performed and a convenience sample of different pharmacy stakeholders was involved: academia (teaching staff at the Institute of Pharmacy), wholesale and retail sale of medicines, hospital pharmacy, representatives of pharmaceutical industry, and other fields (e.g., State Agency of Medicines).

Instead of studying pharmacy students’ perception about the competency-based curriculum, the decision was to involve recently graduated pharmacists who could analyse the pharmacy programme from the point of view of both students and pharmacists. Fourteen evaluators (seven from academia and seven from other pharmacy fields) participated in the assessment. Six of the respondents had graduated recently (1–5 years ago) and eight were experienced specialists.

### 2.3. Data Analysis

As some of the EPCF competences were supported by more than one pharmacy programme subject, evaluators decided to use different types of curriculum elements (subjects, subject areas, and full programme) in the mapping exercise. The subject area was defined by using previously-determined subject groups (Table 2). In the evaluation, all pharmacy programme subjects (obligatory and elective) were used. Detailed description of the pharmacy programme at UT is presented in Appendix A.

Based on the outcomes of curriculum elements and evaluators’ own practical experience, the competency levels were identified according to the personal and patient care competences listed in EPCF. A five-point scale tool, adopted from the Dutch competency standards framework [23] and based on the increase of professional independence, was used as follows:

- 1: Theoretical education; LEVEL OF INDEPENDENCE 1;
- 2: Theoretical education and practical skills; LEVEL OF INDEPENDENCE 2;
- 3: Ability to use theoretical knowledge and practical skills in learning situations; LEVEL OF INDEPENDENCE 3;
- 4: Ability to use theoretical knowledge and practical skills in authentic learning situations (classroom and during pharmacy internship); LEVEL OF INDEPENDENCE 4;
- 5: Ability to use theoretical knowledge and practical skills in practice; LEVEL OF INDEPENDENCE.

The required competency level for recent pharmacy programme graduates at UT could start with a professional independence level 3 and higher. The mean values for 11 competence domains based on the described five-point scale assessment tool were calculated and the results of academia and other pharmacy stakeholders were compared. 

In this article the term “competence” is used to describe knowledge and skills—standards—needed to reach professional performance. The term “competency” describes behaviour and commitment in achieving competences.

## 3. Results

### 3.1. Construction of Curriculum Mapping Matrix

Table 3 shows developed matrix based on the 11 competence domains of the EPCF and the pharmacy programme elements. EPCF personal competences were mostly covered by the full programme with emphasis on some subject areas. The EPCF patient care competences were supported by specific subject areas or specific subjects, and the number of subjects covering these competences increased with study years. The existing pharmacy programme included subjects and subject areas in logical order and, thus, supported the generation of professional competency. More frequent subject areas for reaching the 50 EPCF competences were medical and social sciences, pharmaceutical technology, and pharmacy internship. Medical and social sciences both contributed to 40% and pharmacy internship to 30% of the listed competences. Elective subjects covered personal as well as patient care competences. In some cases, the competence was supported mostly by the elective courses, e.g., the domain “patient education”.

Not all of the EPCF competences were covered with the pharmacy programme subjects. For example: the business and entrepreneurship competences (“ability to identify the need for new services” and “ability to understand a business environment and develop entrepreneurship”) have not been covered by any of the curriculum subjects. The competences about drug registration and marketing; supply chain of medicines, and public health issues have been very briefly taught in different obligatory and elective subjects.

### 3.2. Competencies of Entry-Level Pharmacists

In both academia and other pharmacy stakeholders’ groups the level of personal competencies was assessed higher than patient care competencies (Table 3). In almost all domains, the competency level was perceived to be at a higher level by academia than the pharmacy sector representatives. The latter evaluated personal and patient care competency level as three on the five-point scale. The academia group rated personal competency level higher (four on a five-point scale), but agreed with the pharmacy sector group on patient care competency level. The two groups disagreed the most on the level of personal competencies in the domain “research and industrial pharmacy”. Considerable variations were identified in patient care competency levels in two domains: “drug dose and formulation” and “provision of information and service”.

### 3.3. Curriculum Gaps

Both groups of evaluators described more and less positive aspects of the pharmacy programme. An expansive education was expected to support critical thinking and logical problem solving, large proportion of medical subjects, and the involvement of practicing specialists from different pharmacy fields were emphasized as good basis for contemporary higher education.

Representatives of different pharmacy sectors addressed several issues in the pharmacy programme organization and training methods:

- increase collaboration between different healthcare professions; more integrated training and common courses with medical students;
- support more patient care competencies linking the pharmacy programme with practice on different fields of pharmacy starting already from the first years of studies;
- support the reflection and practical implementation of theoretical knowledge, more broad use of problem-based learning in different subjects;
- introduce business and entrepreneurship subjects to the pharmacy programme as from 2020, pharmacy ownership will be limited only by pharmacy profession in Estonia;
- develop more detailed requirements for pharmacy students and for internship supervisors at community and hospital pharmacies as pharmacy internship plays very important role in implementing of professional competencies.

### 3.4. Usability of EPCF in Curriculum Mapping and Competency Level Evaluation

The EPCF was utilized to identify competencies that have not been covered by the existing pharmacy programme or competencies that were not sufficiently supported by existing subjects. As the EPCF has been designed with a focus on professional competencies required in community pharmacy, this could, to some extent, impede using this type of tool for evaluation of the curriculum without specialization. This kind of training focuses on providing as broad professional knowledge as possible and does not concentrate in detail only on patient care competencies. For competency level evaluation, a proposal was made to use the five-step Dutch competency standards framework because Miller’s ‘pyramid’ (knows, knows how, shows how, and does) does not provide a detailed description of professional knowledge and independence of pharmacists on different competency levels. To address more country-specific needs, other evaluation methods could be considered or developed in future.

## 4. Discussion

This was the first time the use a competency-based model has been applied for the evaluation of the pharmacy programme at the UT. The assessment provided important information and it is in line with the recommendations presented by an international accreditation team of the Medicine Study Programme Group at UT in 2014 [24]. Both evaluations stressed the need for integrated and novel training methods, for self-reflection and analysis of acquired knowledge by students and for more frequent collaboration between practicing specialists so theoretical knowledge could be implemented into practice better. 

Gradual implementation of medical subjects to the pharmacy programme was planned to increase professional competency of pharmacists as healthcare professionals. Based on the EPCF evaluation, the medical subjects of the existing pharmacy programme might not provide sufficient support to patient care competencies in terms of practical usability. However, the description of professional competencies for pharmacists and assistant pharmacists had been missing for the past two decades in Estonia and this situation was not conductive to providing practice-linked training. In November 2016, the occupational qualification standards for pharmacists and assistant pharmacists were approved in Estonia [25]. The standards enable clear identification of professional roles within the pharmacy profession and provide detailed descriptions of required competencies. This kind of supporting information will be vital for the advancement of pharmacy education in Estonia in future.

Representatives of the academia and other pharmacy stakeholders gave different scores to EPCF personal and patient care competencies level. Level of personal competencies was evaluated with a higher score than level of patient care competencies. As many of personal competences were seen as covered by the full pharmacy programme, the level of evaluated competencies could be more speculative than in the case of patient care competences where specific subjects were listed to support the particular competence. 

Although the common agreement was to analyse the existing pharmacy programme, the competency evaluation could be conducted on various levels: for academia as knowledge-delivered level and for other pharmacy stakeholders as mix of perceived (viewpoint of students) and realized (viewpoint of practicing specialists and employers) levels. For academia, the evaluation was based on assumptions about the use of theoretical knowledge in practice. Representatives of other pharmacy sectors were expecting not only theoretically competent specialists, but somebody who could perform and fluently use acquired professional knowledge in their specific field of pharmacy. The delivery of diverse educational content might be the reason for surprisingly large score variations given by academia and other pharmacy stakeholders to patient care competencies: “drug dose and formulation” and “provision of information and service”. Both domains describe core competencies of the pharmacy profession and have to be covered by any type of pharmacy curriculum. However, training methods play an important role in the use of acquired knowledge. Case- and problem-based learning could assure more practical use of theoretical knowledge and help future specialists to settle into working life more easily. Pharmacists planning to work in particular areas could have the possibility of constructing a description or list of competencies that are of importance and could assist in their professional performance. This type of differential approach is described in the National Competency Standards Framework for Pharmacists in Australia and would help to elucidate what competencies have to be developed more at CPD courses [19]. 

The development and implementation of a competency based pharmacy curriculum is a long process and requires common understanding of the higher education institution, other partners in the pharmacy sector and governmental institutions about the demand for considerable change in pharmacy education. 

The UT has recently paid a lot of attention to the improvement of teaching and learning quality and published principles of Good Practice of Learning and Good Practice of Teaching, which is a part of the university's good practices. The new practice stimulates students and university staff members to be motivated to teach and learn. In addition, students are encouraged to self-reflect and analyse their studies, which is very important feedback in outcome-oriented professional education [26,27]. Good Practice of Teaching encourages development of teaching communities and emphasises the following principles: excellent teaching is learning centred, based on a scientific way of thinking and cooperation, supports creativity and entrepreneurship, leads to self-analysis, and supports individual development and links learning to real life [27].

In addition to the redesigning of the teaching and learning practices, it is necessary to assure conditions for the provision of competency based clinical practice. The development of professional competency is a continuous process involving both under- and post-graduate education.

Limitations

For all evaluators, curriculum development was not their field of expertise. In the future, it would be necessary to consult with curriculum development specialists and education scientists at the UT about curriculum organization and novel training methods. Additionally, more pharmacy stakeholders have to be involved in discussions and the decision-making process in order to identify the needs for pharmacy competence teaching in Estonia.

## 5. Conclusions

The EPCF-based mapping exercise of the pharmacy programme at UT provided useful information regarding professional competencies of entry-level pharmacists who have completed traditional pharmacy curriculum. Most of the EPCF competence domains were covered by subjects, subject areas, or the full pharmacy programme. However, to assure independent and responsible patient-centred professional practice, personal and patient care competency levels at graduation could be higher. Representatives of academia and other pharmacy stakeholders concluded that the existing pharmacy programme is designed to provide broad theoretical knowledge, but more efficient training methods should be implemented and practicing specialists from different fields of pharmacy should participate in the teaching process to link theory with practice more efficiently. 

Additional research and continuous collaboration within the pharmacy sector is important in order to understand what would the most applicable way to move towards competency-based pharmacy training in Estonia.

## Figures and Tables

**Table 1:** Comparison of seven subject areas in pharmacy programmes in EU and UT, Estonia.

Subject Area	Proportion of Subject Areas in Pharmacy Programmes EU Main %	Proportion of Subject Areas in the Pharmacy Programme (UT), Estonia %
Chemical sciences	26	21
Physics, mathematics	6	4
Biological sciences	11	2
Pharmaceutical technology	16	21
Medical sciences	28	39
Law and social sciences	7	10
Generic subjects, traineeship	6	3

**Table 2:** Distribution of subject groups within first four study years in the pharmacy programme (UT).

Study Year	Drug Analysis	Pharmaceutical Technology	Medical Sciences and Patient Care Subjects	Social Sciences
**First**	Inorganic and analytical chemistry	-	Anatomy, physiology, medical microbiology	Latin, pharmaceutical terminology, history of pharmacy
**Second**	Analytical, bioorganic and pharmaceutical chemistry	Pharmaceutical excipients	Physiology, pathophysiology, clinical microbiology, medical biochemistry, genetics, primary care medicine	Bioethics
**Third**	Pharmacognosy, pharmaceutical chemistry	Pharmaceutical technology, biotechnology	Laboratory medicine, pharmacology	-
**Fourth**	Metabolism of active substances	Physical pharmacy, biopharmacy	Immunology, pharmacotherapy, first aid, drug toxicology, clinical pharmacology, clinical pharmacy,	Pharmaco- epidemiology and pharmaco-economics,social pharmacy, and drug safety

**Table 3:** EPCF and the pharmacy programme (UT) based curriculum mapping matrix and mean values of competency levels assessed by representatives of academia and other pharmacy stakeholders.

Competence Domains	Specific Subjects	Subject Area/-s	Full Programme	Mean Competency Level *, Academia	Mean Competency Level *, Other Pharmacy Stakeholders
**Personal competences**					
1. Learning and knowledge	NA **	Medical and social sciences, pharmacy internship	Yes	4.0	3.4
2. Values	NA	Social sciences, pharmacy internship	Yes	4.4	3.7
3. Communication and organizational skills	NA	Social sciences, pharmacy internship	Yes	3.8	3.1
4. Research and industrial pharmacy	Yes	Pharmaceutical technology, drug analysis	Yes, only one competence	4.0	2.5
**Personal competence domains 1–4 mean value**				**4.1**	**3.2**
**Patient care competences**					
5. Patient consultation and assessment	Yes	Medical sciences	NA	3.3	3.2
6. Need for drug treatment	Yes	Medical sciences	NA	3.0	3.0
7. Drug interactions	Yes	Medical sciences, pharmaceutical technology	NA	3.0	3.2
8. Drug dose and formulation	Yes	Pharmaceutical technology, medical sciences	NA	4.2	3.0
9. Patient education	Yes	No	NA	4.0	3.7
10. Provision of information and service	Yes	Medical and social sciences, pharmaceutical technology, pharmacy internship	NA	4.7	3.5
11. Monitoring of drug therapy	Yes	Medical and social sciences, pharmaceutical technology, pharmacy internship	NA	3.2	2.4
**Patient care competence domains 5–11 mean value**				**3.6**	**3.1**

* Five-point competency level evaluation scale: 1-theoretical education and 5-ability to use independently theoretical knowledge and practical skills in practice; ** NA—not applicable.

## Appendix A

**Table A1:** Content of the curriculum Pharmacy for entrants at the University of Tartu, Estonia, of 2016/2017.

Pharmacy (300 ECTS)				
***1. Compulsory subjects (224 ECTS)***				
*1.1 First course compulsory subjects (51 ECTS)*				
	ARFA.02.099	Analytical Chemistry	12	ECTS
	ARAN.01.034	Anatomy	5	ECTS
	ARFS.01.023	Biophysics	4	ECTS
	LOKT.00.004	General and Inorganic Chemistry	6	ECTS
	ARFS.01.063	Human Physiology	10	ECTS
	ARFA.01.071	Introduction and History of Pharmacy	3	ECTS
	FLKE.05.009	Latin in Pharmacy	3	ECTS
	ARMB.00.001	Medical Microbiology	6	ECTS
	ARFA.02.098	Pharmaceutical Terminology	3	ECTS
	ARFA.01.072	Pharmacognosy I	3	ECTS
	MTMS.01.085	Statistical Analysis	4	ECTS
*1.2 Second course compulsory subjects (52 ECTS)*				
	ARFA.02.099	Analytical Chemistry	12	ECTS
	ARFA.02.108	Bioethics	4	ECTS
	ARBK.01.042	Bioorganic Chemistry	5	ECTS
	ARMB.01.053	Clinical Microbiology	2	ECTS
	ARMP.01.030	Genetics	3	ECTS
	ARFS.01.063	Human Physiology	10	ECTS
	ARBK.01.043	Medical Biochemistry	5	ECTS
	ARMP.03.031	Pathophysiology	6	ECTS
	ARFA.02.109	Pharmaceutical Chemistry I	9	ECTS
	ARFA.02.103	Pharmaceutical Excipients	5	ECTS
	ARPO.00.020	Primary Care Medicine	5	ECTS
*1.3 Third course compulsory subjects (52 ECTS)*				
	ARMP.03.035	Biotechnology	3	ECTS
	ARSK.03.017	Introduction to the Laboratory Medicine	2	ECTS
	ARFA.02.112	Pharmaceutical Chemistry II	8	ECTS
	ARFA.02.113	Pharmaceutical Technology	22	ECTS
	ARFA.01.105	Pharmacognosy II	7	ECTS
	ARFA.01.077	Pharmacognosy III	4	ECTS
	ARFR.02.051	Pharmacology	11	ECTS
*1.4 Fourth course compulsory subjects (51 ECTS)*				
	ARFA.02.144	Biopharmaceutics	5	ECTS
	ARFR.03.018	Clinical Pharmacology	2	ECTS
	ARFA.01.104	Clinical Pharmacy	3	ECTS
	ARFR.02.034	Drug Toxicology	3	ECTS
	ARKI.01.004	First Aid	3	ECTS
	ARMP.02.016	Immunology	2	ECTS
	ARFA.02.123	Metabolism of Active Substances	3	ECTS
	ARFA.02.113	Pharmaceutical Technology	22	ECTS
	ARFA.01.059	Pharmacoepidemiology and Pharmacoeconomics	3	ECTS
	ARFR.02.052	Pharmacotherapy	5	ECTS
	ARFA.02.122	Physical Pharmacy	6	ECTS
	ARFA.01.082	Social Pharmacy and Drug Safety	11	ECTS
*1.5 Fifth course (18 ECTS)*				
	ARFA.02.040	Designing of Research Work	1	ECTS
	ARFA.02.044	Pharmaceutical Commodities	4	ECTS
	ARFA.00.057	Research Work	6	ECTS
or	ARFS.01.076	Diploma Work	6	ECTS
or	ARBK.01.048	Research Work	6	ECTS
or	ARMB.01.059	Diploma Work	6	ECTS
or	ARFR.02.061	Diploma Work	6	ECTS
or	ARAN.00.040	Research Project	6	ECTS
seminar of research (7 ECTS) or				
	ARFA.02.117	Research Seminar on Pharmaceutical Chemistry	7	ECTS
	ARFA.02.116	Research Seminar on Pharmaceutical Technology	7	ECTS
	ARFA.01.078	Research Seminar on Pharmacognosy	7	ECTS
	ARFA.01.080	Research Seminar on Social Pharmacy	7	ECTS
	ARFA.01.096	Research Seminars on Clinical Pharmacy	7	ECTS
	ARFA.02.118	Research Seminars on History of Pharmacy	7	ECTS
	ARFS.01.075	Research Workshop of Physiology	7	ECTS
	ARAN.02.027	Seminar on Histology	7	ECTS
	ARBK.01.047	Seminars of Research in Medical Biochemistry	7	ECTS
	ARMB.01.060	Seminars of Research in Medical Microbiology	7	ECTS
	ARFR.02.060	Seminars of Research in Pharmacology	7	ECTS
***2. Elective courses (22 ECTS)***				
*2.1 First course elective subjects (6 ECTS)*				
	FLKE.03.151	Estonian for Students of Medicine, on the Basis of Russian, Level B1 >B2	3	ECTS
	ARFA.02.080	Exercises and Problems in Pharmaceutical Analysis	1	ECTS
	ARLA.01.034	Foetal and Neonatal Physiology and Behaviour, Breastfeeding	2	ECTS
	ARSK.00.009	Gerontology. Introduction (web-based)	3	ECTS
	FLFI.00.002	Introduction to Philosophy	2	ECTS
	ARFA.01.106	Pharmacist in pharmacy system	2	ECTS
	ARTH.04.023	Prevention of HIV/AIDS and HIV Positive Patient	2	ECTS
	ARTH.04.022	Principles of Sexuality Education	3	ECTS
	ARFA.02.145	Seminars in Pharmaceutical Sciences I	1	ECTS
	ARTH.04.024	Smoking and Health	1	ECTS
	LOKT.01.017	Solutions	2	ECTS
	ARSI.01.015	Spectacles, Contact Lenses and Refractive Surgery	1	ECTS
	ARLA.01.033	Why and to Which Factors We are Allergic?	2	ECTS
*2.2 Second course elective subjects (5 ECTS)*				
	ARBK.01.021	Basic Knowledge for the Research in Medical Biochemistry (I)	2	ECTS
	ARMP.03.019	Basic Research in Pathophysiology I	6	ECTS
	ARMP.03.020	Basic Research in Pathophysiology II	6	ECTS
	ARFA.02.080	Exercises and Problems in Pharmaceutical Analysis	1	ECTS
	ARLA.01.034	Foetal and Neonatal Physiology and Behaviour, Breastfeeding	2	ECTS
	ARSK.00.009	Gerontology. Introduction (web-based)	3	ECTS
	ARFA.02.138	Interdisciplinary Seminars in Pharmacy	2	ECTS
	ARFS.01.005	Neurophysiology of Pain	2	ECTS
	ARFA.01.106	Pharmacist in pharmacy system	2	ECTS
	ARTH.04.023	Prevention of HIV/AIDS and HIV Positive Patient	2	ECTS
	ARTH.04.022	Principles of Sexuality Education	3	ECTS
	ARFA.02.019	Propaedeutical Training	3	ECTS
	ARFA.02.081	Radiopharmaceuticals	1	ECTS
	ARFA.02.145	Seminars in Pharmaceutical Sciences I	1	ECTS
	ARTH.04.024	Smoking and Health	1	ECTS
	ARSI.01.015	Spectacles, Contact Lenses and Refractive Surgery	1	ECTS
	ARFA.02.128	Student Research in Pharmacy I	3	ECTS
	ARFA.02.135	Student Research in Pharmacy II	6	ECTS
	ARLA.01.033	Why and to Which Factors We are Allergic?	2	ECTS
*2.3 Third course elective subjects (5 ECTS)*				
	ARBK.01.035	Basic Knowledge for the Research in Medical Biochemistry (II)	5	ECTS
	ARMP.03.019	Basic Research in Pathophysiology I	6	ECTS
	ARMP.03.020	Basic Research in Pathophysiology II	6	ECTS
	ARFA.02.124	Chemistry of Vitamins	2	ECTS
	ARTH.01.080	Environmental and Occupational Toxicology	3	ECTS
	ARFA.02.080	Exercises and Problems in Pharmaceutical Analysis	1	ECTS
	ARLA.01.034	Foetal and Neonatal Physiology and Behaviour, Breastfeeding	2	ECTS
	ARSK.00.009	Gerontology. Introduction (web-based)	3	ECTS
	ARFA.02.126	Granulation and Tableting Technology	2	ECTS
	ARTH.04.018	Health Promotion	2	ECTS
	ARFA.02.138	Interdisciplinary Seminars in Pharmacy	2	ECTS
	ARFS.01.005	Neurophysiology of Pain	2	ECTS
	ARMP.03.025	Pathophysiology of Stress	1.5	ECTS
	ARFA.01.106	Pharmacist in pharmacy system	2	ECTS
	ARTH.04.023	Prevention of HIV/AIDS and HIV Positive Patient	2	ECTS
	ARTH.04.022	Principles of Sexuality Education	3	ECTS
	ARFA.02.081	Radiopharmaceuticals	1	ECTS
	ARFA.02.145	Seminars in Pharmaceutical Sciences I	1	ECTS
	ARTH.04.024	Smoking and Health	1	ECTS
	ARSI.01.015	Spectacles, Contact Lenses and Refractive Surgery	1	ECTS
	ARFA.02.128	Student Research in Pharmacy I	3	ECTS
	ARFA.02.135	Student Research in Pharmacy II	6	ECTS
	ARFA.02.130	Student Research in Pharmacy III	6	ECTS
	ARFA.02.133	Student Research in Pharmacy IV	6	ECTS
	ARFA.02.015	Synthesis of Active Substances	2	ECTS
	ARFA.02.140	Vibrational Spectroscopy- Different Applications in Pharmaceutical Drug Development	1	ECTS
	ARTH.01.014	Water and Health	2	ECTS
	ARLA.01.033	Why and to Which Factors We are Allergic?	2	ECTS
	ARTH.01.040	Work Stress and Health	2	ECTS
*2.4 Fourth course elective subjects (6 ECTS)*				
	AROT.00.030	Andragogy and Higher Education	3	ECTS
	AROT.00.033	Applications of Developmental Psychology in Nursing	3	ECTS
	ARMP.03.019	Basic Research in Pathophysiology I	6	ECTS
	ARMP.03.020	Basic Research in Pathophysiology II	6	ECTS
	ARTH.01.080	Environmental and Occupational Toxicology	3	ECTS
	ARFA.02.080	Exercises and Problems in Pharmaceutical Analysis	1	ECTS
	ARLA.01.034	Foetal and Neonatal Physiology and Behaviour, Breastfeeding	2	ECTS
	ARSK.00.009	Gerontology. Introduction (web-based)	3	ECTS
	ARFA.02.126	Granulation and Tableting Technology	2	ECTS
	AROT.00.036	Health Care System and Policy for Nursing Managers	3	ECTS
	ARTH.04.018	Health Promotion	2	ECTS
	ARFA.02.138	Interdisciplinary Seminars in Pharmacy	2	ECTS
	ARAR.00.001	Medical Law	2	ECTS
	ARFA.02.134	Minimally Invasive Medical Technology	2	ECTS
	ARFS.01.005	Neurophysiology of Pain	2	ECTS
	ARFA.02.132	Pharmaceutical Film and Nano-coatings	2	ECTS
	ARFA.02.141	Pharmaceutical nanotechnology	4	ECTS
	ARFA.01.106	Pharmacist in pharmacy system	2	ECTS
	AROT.01.013	Planning and Designing of Developmental Projects in Nursing	3	ECTS
	ARTH.04.023	Prevention of HIV/AIDS and HIV Positive Patient	2	ECTS
	ARTH.04.022	Principles of Sexuality Education	3	ECTS
	ARFA.02.081	Radiopharmaceuticals	1	ECTS
	ARFA.02.145	Seminars in Pharmaceutical Sciences I	1	ECTS
	ARTH.04.024	Smoking and Health	1	ECTS
	ARFA.01.060	Social Pharmacy in the Professional Literature and Internet	2	ECTS
	ARSI.01.015	Spectacles, Contact Lenses and Refractive Surgery	1	ECTS
	ARFA.02.135	Student Research in Pharmacy II	6	ECTS
	ARFA.02.130	Student Research in Pharmacy III	6	ECTS
	ARFA.02.133	Student Research in Pharmacy IV	6	ECTS
	ARFA.02.137	Student Research in Pharmacy V	6	ECTS
	ARFA.02.015	Synthesis of Active Substances	2	ECTS
	ARSK.00.027	Vaccine and Immunization Education	3	ECTS
	ARFA.02.140	Vibrational Spectroscopy- Different Applications in Pharmaceutical Drug Development	1	ECTS
	ARTH.01.014	Water and Health	2	ECTS
	ARLA.01.033	Why and to Which Factors We are Allergic?	2	ECTS
	ARTH.01.040	Work Stress and Health	2	ECTS
***3. Pharmacy Practice (37 ECTS)***				
	ARFA.02.071	Pharmacy Practice	37	ECTS
***4. Final Exam (5 ECTS)***				
	ARFA.02.060	Graduation Examination	5	ECTS
***5. Optional courses (12 ECTS)***				

## Appendix B

**Table A2:** The European pharmacy competences framework.

	Domains and compenetnes
	**Domain 1: Personal competences—learning and knowledge**
1	Ability to identify learning needs and to learn independently (including continuous professional development, CPD).
2	Ability to apply logic to problem solving.
3	Ability to critically appraise relevant knowledge and to summarise the key points.
4	Ability to evaluate scientific data in line with current scientific and technological knowledge.
5	Ability to apply preclinical and clinical evidence-based medical science to pharmaceutical practice.
6	Ability to apply current knowledge of relevant legislation and codes of pharmacy practice.
	**Domain 2: Personal competences—values**
7	A professional approach to tasks and human relations.
8	Ability to maintain confidentiality.
9	Ability to take full responsibility for patient care.
10	Ability to inspire the confidence of others in one’s actions and advise.
11	Knowledge of appropriate legislation and of ethics.
	**Domain 3: Personal competences—communication and organisational skills**
12	Ability to communicate effectively—both oral and written—in the locally relevant language.
13	Ability to effectively use information technology.
14	Ability to work effectively as part of a team.
15	Ability to implement general legal requirements that impact upon the practice of pharmacy (e.g., health and safety legislation, employment law).
16	Ability to contribute to the training of staff.
17	Ability to manage risk and quality of service issues.
18	Ability to identify the need for new services.
19	Ability to understand a business environment and develop entrepreneurship.
	**Domain 4: Personal competences—research and industrial pharmacy**
20	Knowledge of design, synthesis, isolation, characterisation and biological evaluation of active substances.
21	Knowledge of good manufacturing practice and of good laboratory practice.
22	Knowledge of European directives on qualified persons.
23	Knowledge of drug registration, licensing and marketing.
24	Knowledge of the importance of research in pharmaceutical development and practice.
	**Domain 5: Patient care competences—patient consultation and assessment**
25	Ability to interpret basic medical laboratory tests.
26	Ability to perform appropriate diagnostic tests e.g., measurement of blood pressure or blood sugar.
27	Ability to recognise when referral to another member of the healthcare team is needed.
	**Domain 6: Patient care competences—need for drug treatment**
28	Ability to retrieve and interpret information on the patient’s clinical background.
29	Ability to compile and interpret a comprehensive drug history for an individual patient.
30	Ability to identify non-adherence to medicine therapy and make an appropriate intervention.
31	Ability to advise to physicians on the appropriateness of prescribed medicines and—in some cases—to prescribe medication.
	**Domain 7: Patient care competences—drug interactions**
32	Ability to identify and prioritise drug-drug interactions and advise appropriate changes to medication.
33	Ability to identify and prioritise drug-patient interactions, including those that prevent or require the use of a specific drug, based on pharmaco-genetics, and advise on appropriate changes to medication.
34	Ability to identify and prioritise drug-disease interactions (e.g., NSAIDs in heart failure) and advise on appropriate changes to medication.
	**Domain 8: Patient care competences—drug dose and formulation**
35	Knowledge of the bio-pharmaceutical, pharmacodynamic and pharmacokinetic activity of a substance in the body.
36	Ability to recommend interchangeability of drugs based on in-depth understanding and knowledge of bioequivalence, bio-similarity and therapeutic equivalence of drugs.
37	Ability to undertake a critical evaluation of a prescription ensuring that it is clinically appropriate and legally valid.
38	Knowledge of the supply chain of medicines thus ensuring timely flow of quality drug products to the patient.
39	Ability to manufacture medicinal products that are not commercially available.
	**Domain 9: Patient care competences—patient education**
40	Ability to promote public health in collaboration with other professionals within the healthcare system.
41	Ability to provide appropriate lifestyle advice to improve patient outcomes (e.g., advice on smoking, obesity, etc.).
42	Ability to use pharmaceutical knowledge and provide evidence-based advice on public health issues involving medicines.
	**Domain 10: Patient care competences—provision of information and service**
43	Ability to use effective consultations to identify the patient’s need for information.
44	Ability to provide accurate and appropriate information on prescription medicines.
45	Ability to provide evidence-based support for patients in selection and use of non- prescription medicines.
	**Domain 11: Patient care competences—monitoring of drug therapy**
46	Ability to identify and prioritise problems in the management of medicines in a timely and effective manner and so ensure patient safety.
47	Ability to monitor and report Adverse Drug Events and Adverse Drug Reactions (ADEs and ADRs) to all concerned, in a timely manner, and in accordance with current regulatory guidelines on Good Pharmacovigilance Practices (GVPs).
48	Ability to undertake a critical evaluation of prescribed medicines to confirm that current clinical guidelines are appropriately applied.
49	Ability to monitor patient care outcomes to optimise treatment in collaboration with the prescriber.
50	Ability to contribute to the cost effectiveness of treatment by collection and analysis of data on medicines use.
